# A Rare Late Complication of Port Catheter Implantation: Embolization of the Catheter

**DOI:** 10.4274/tjh.2017.0134

**Published:** 2018-05-25

**Authors:** Işık Odaman Al, Cengiz Bayram, Gizem Ersoy, Kazım Öztarhan, Alper Güzeltaş, Taner Kasar, Ezgi Uysalol, Başak Koç, Ali Ayçiçek, Nihal Özdemir

**Affiliations:** 1 University of Health Sciences, İstanbul Kanuni Sultan Süleyman Training and Research Hospital, Clinic of Pediatric Hematology and Oncology, İstanbul, Turkey; 2 University of Health Sciences, İstanbul Kanuni Sultan Süleyman Training and Research Hospital, Clinic of Pediatric Cardiology, İstanbul, Turkey; 3 University of Health Sciences, İstanbul Mehmet Akif Ersoy Thoracic and Cardiovascular Surgery Training and Research Hospital, Clinic Pediatric Cardiology, İstanbul, Turkey

**Keywords:** Acute lymphoblastic leukemia, Catheter, Complication

**To the Editor,**

Children with cancer need long-term venous access due to the long duration of therapy. Long-term totally implantable port devices (TIPDs) are widely used in these patients for administration of chemotherapeutic agents, parenteral nutrition, fluids, and blood products [1,2]. Fracture and embolism of TIPDs are rare complications but may cause serious results and mortality, including pulmonary artery embolism, sepsis, arrhythmias, and perforation of the caval vein [3,4,5]. Herein, we present a 9-year-old male patient with pre-B acute lymphoblastic leukemia who was admitted to the outpatient pediatric hematology and oncology clinic at the 13^th^ month of maintenance therapy due to new onset of non-flushing catheter. The patient had no other complaints. On posterior anterior chest X-ray, the catheter was found to be disconnected from its reservoir (Figure 1). Echocardiography and thorax computed tomography angiography of the patient revealed the embolization of the catheter to the left pulmonary artery (Figure 2). The embolized catheter was removed using an interventional endovascular procedure under general anesthesia through the femoral vein by an interventional cardiologist (Figure 3). Our case report highlights a rarely encountered complication of TIPDs, which may be undiagnosed due to its rarity and lack of symptoms in some patients, leading to serious complications.

## Figures and Tables

**Figure 1:**
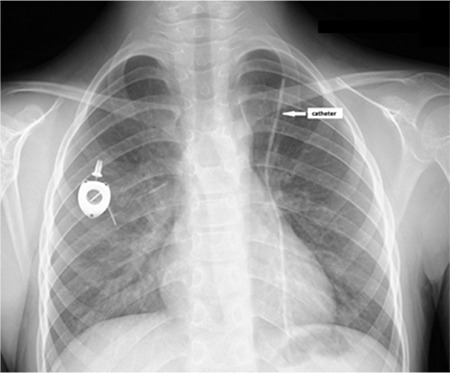
Chest X-ray showing disconnection of the catheter from its reservoir.

**Figure 2:**
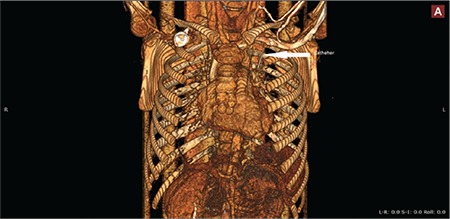
Thorax computed tomography angiography of the patient showing the embolization of the catheter to the left pulmonary artery.

**Figure 3:**
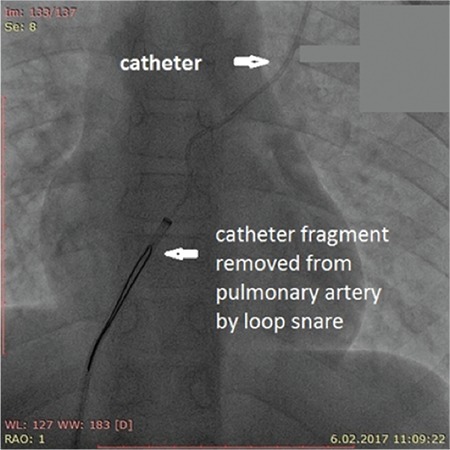
Removal of the catheter with an interventional endovascular procedure from pulmonary artery.
